# Safety and effectiveness of SGLT2 inhibitors in a UK population with type 2 diabetes and aged over 70 years: an instrumental variable approach

**DOI:** 10.1007/s00125-024-06190-9

**Published:** 2024-06-05

**Authors:** Laura M. Güdemann, Katie G. Young, Nicholas J. M. Thomas, Rhian Hopkins, Robert Challen, Angus G. Jones, Andrew T. Hattersley, Ewan R. Pearson, Beverley M. Shields, Jack Bowden, John M. Dennis, Andrew P. McGovern, on behalf of the MASTERMIND consortium

**Affiliations:** 1 Institute of Biomedical & Clinical Science, University of Exeter Medical School, Exeter, UK; 2 Division of Molecular & Clinical Medicine, Ninewells Hospital and Medical School, University of Dundee, Dundee, UK

**Keywords:** Causal analysis, Effectiveness, Older adults, Safety, SGLT2 inhibitors

## Abstract

**Aims/hypothesis:**

Older adults are under-represented in trials, meaning the benefits and risks of glucose-lowering agents in this age group are unclear. The aim of this study was to assess the safety and effectiveness of sodium–glucose cotransporter 2 inhibitors (SGLT2i) in people with type 2 diabetes aged over 70 years using causal analysis.

**Methods:**

Hospital-linked UK primary care data (Clinical Practice Research Datalink, 2013–2020) were used to compare adverse events and effectiveness in individuals initiating SGLT2i compared with dipeptidyl peptidase-4 inhibitors (DPP4i). Analysis was age-stratified: <70 years (SGLT2i *n*=66,810, DPP4i *n*=76,172), ≥70 years (SGLT2i *n*=10,419, DPP4i *n*=33,434). Outcomes were assessed using the instrumental variable causal inference method and prescriber preference as the instrument.

**Results:**

Risk of diabetic ketoacidosis was increased with SGLT2i in those aged ≥70 (incidence rate ratio compared with DPP4i: 3.82 [95% CI 1.12, 13.03]), but not in those aged <70 (1.12 [0.41, 3.04]). However, incidence rates with SGLT2i in those ≥70 was low (29.6 [29.5, 29.7]) per 10,000 person-years. SGLT2i were associated with similarly increased risk of genital infection in both age groups (incidence rate ratio in those <70: 2.27 [2.03, 2.53]; ≥70: 2.16 [1.77, 2.63]). There was no evidence of an increased risk of volume depletion, poor micturition control, urinary frequency, falls or amputation with SGLT2i in either age group. In those ≥70, HbA_1c_ reduction was similar between SGLT2i and DPP4i (−0.3 mmol/mol [−1.6, 1.1], −0.02% [0.1, 0.1]), but in those <70, SGLT2i were more effective (−4 mmol/mol [4.8, −3.1], −0.4% [−0.4, −0.3]).

**Conclusions/interpretation:**

Causal analysis suggests SGLT2i are effective in adults aged ≥70 years, but increase risk for genital infections and diabetic ketoacidosis. Our study extends RCT evidence to older adults with type 2 diabetes.

**Graphical Abstract:**

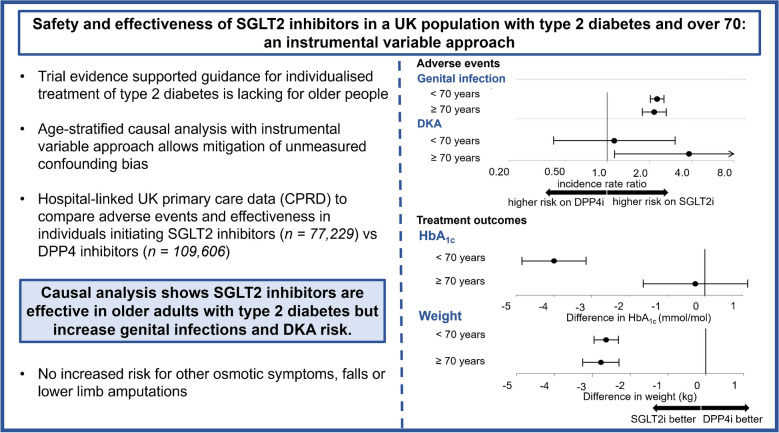

**Supplementary Information:**

The online version of this article (10.1007/s00125-024-06190-9) contains peer-reviewed but unedited supplementary material.


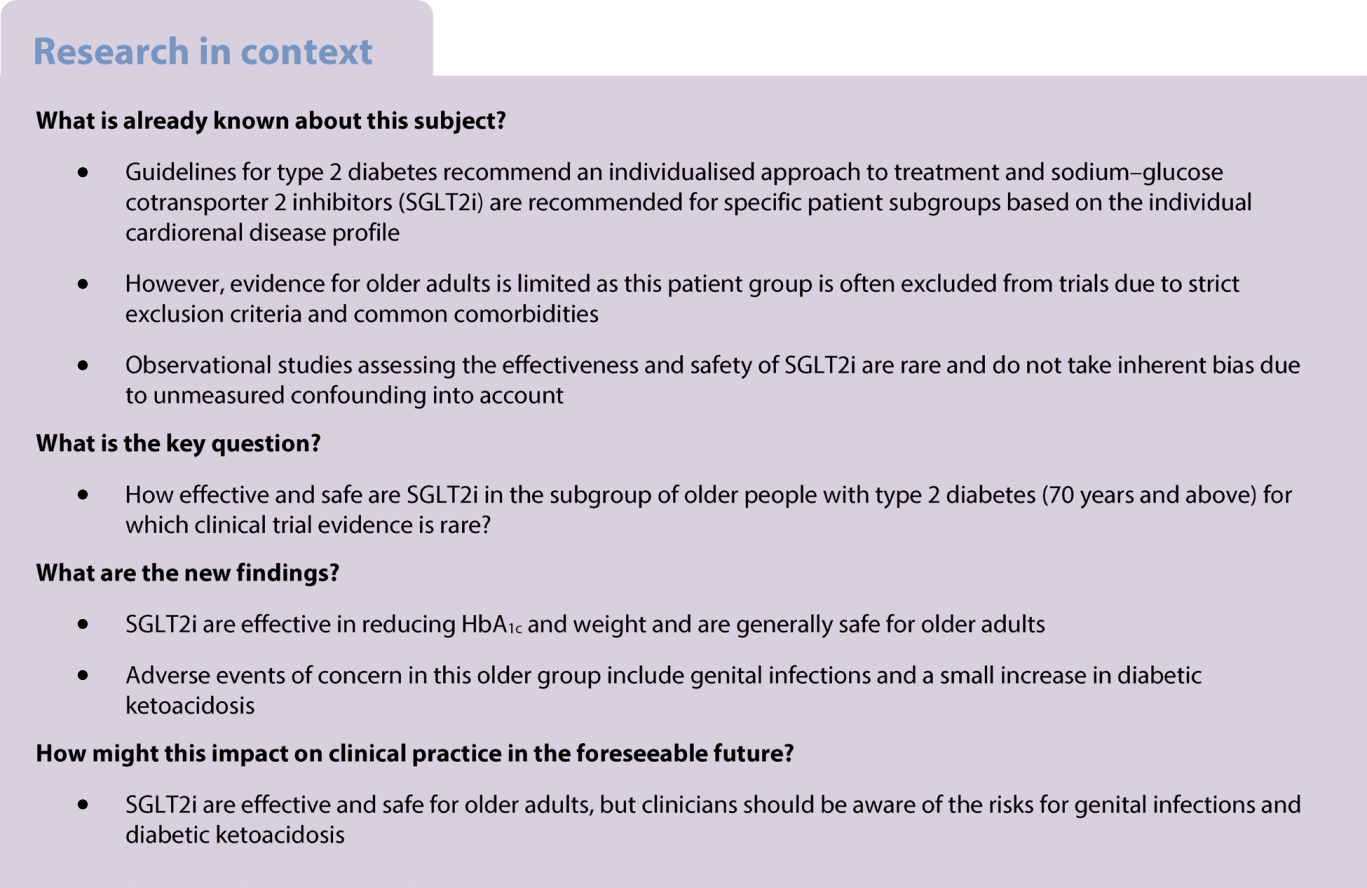


## Introduction

Current type 2 diabetes guidelines recommend an individualised approach to treatment that takes into account preferences, comorbidities, risks from polypharmacy, and the likelihood of long-term benefit from interventions [1, 2], but clear guidance on therapeutic strategies for the management of type 2 diabetes in older adults is limited [3]. For older adults, specific treatment considerations are likely to be needed, due to increased comorbidities, age-related changes in physiology and pharmacodynamics, as well as possible increased propensity to adverse medication effects.

Under current guidelines, a large proportion of older people with type 2 diabetes would be recommended sodium-glucose cotransporters 2 inhibitors (SGLT2i) due to their cardiorenal benefits, and irrespective of their glycaemic control [1, 4]. SGLT2i have well described benefits, particularly cardiorenal benefits and the promotion of weight loss [5–8], but also possible risks, which may limit their use for older people [3]. Well-established risks of SGLT2i are genital infections and, due to their mode of action, volume depletion is possible [6, 9]. These side effects could be of particular concern for older adults where incontinence, dehydration and dizziness could have more severe consequences compared with a younger population [10–13]. Additionally, dehydration or dizziness can also lead to falls in older people [14]. Further adverse events (AEs) of concern of SGLT2i are lower limb amputations [9]. Reports of possible association of SGLT2i and diabetic ketoacidosis (DKA) has prompted the U.S. Food and Drug Administration (FDA) [15] and the European Medicines Agency (EMA) [16] to issue warnings. Older people may also present with more frequent acute complications, such as infections, which are additional risk factors of DKA [17].

In order to develop targeted guidelines for the management of type 2 diabetes in older adults, evidence on risks and benefits of treatments in this age group is needed [3]. However, older people are under-represented in RCTs and caution is needed when extrapolating RCT evidence for this group [3, 18]. Observational studies of the older type 2 diabetes population have the potential to provide insights that are not provided by RCTs. Previous post hoc RCT analyses [13, 19–21] have examined risks in older adults, but have very small sample size for older people with type 2 diabetes, and therefore might suffer from outlier effects [13]. Also, without detailed data on characteristics, comorbidities and concomitant medications, the results from observational studies may be affected by unmeasured confounding which can bias treatment effect results [14].

We therefore aimed to examine the relative risks and benefits of SGLT2i in older people compared with dipeptidyl peptidase-4 inhibitors (DPP4i) using large-scale routine primary and linked secondary care data. We employ an instrumental variable approach, exploiting systematic variation in practitioners’ prescribing preference as the instrument, to estimate the impact of receiving SGLT2i compared with DPP4i on a range of AEs and important treatment outcomes, analogous to an RCT.

## Methods

### Study design and participants

**Fig. 1:**
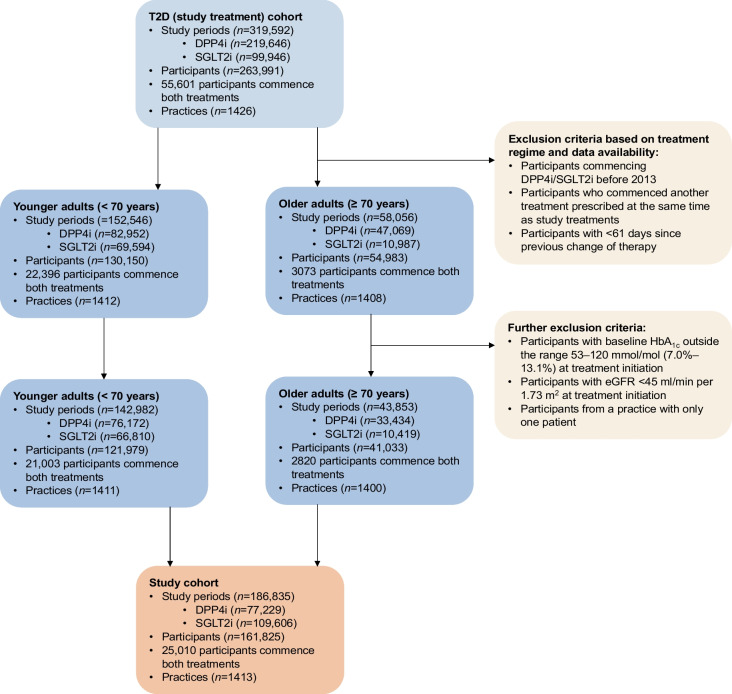
Flow chart of study cohort selection, stratified by age

In this retrospective cohort study, UK routine primary care data were accessed from Clinical Practice Research Datalink (CPRD) Aurum (October 2020 download). CPRD is a UK representative sample covering approximately 13% of the population in England [22]. CPRD Aurum was linked to Hospital Episode Statistics (HES), Office for National Statistics (ONS) death registrations and individual-level Index of Multiple Deprivation (IMD). Individuals with type 2 diabetes were identified according to a previously published protocol [23] based on the presence of a diagnostic code for diabetes and the prescription of one or more glucose-lowering medications. Type 1 diabetes and other types of diabetes were excluded [23]. The analysis included new users of SGLT2i (canagliflozin, dapagliflozin, empagliflozin, ertugliflozin), commencing treatment after 1 January 2013 and with an identifiable date of type 2 diabetes diagnosis. The comparison cohort was new users of DPP4i (alogliptin, linagliptin, sitagliptin, saxagliptin, vildagliptin), as these agents represent the most commonly prescribed drug class after metformin in the UK, and have no known association with the SGLT2i-associated AEs of interest evaluated in this study. All available follow-up data were considered in the analysis up to the point of data extraction. Individuals with a baseline HbA_1c_ outside the range 53–120 mmol/mol (7%–13.1%) were excluded from the analysis, reflecting the threshold for glucose-lowering medication initiation in clinical guidelines and clinical guidelines for severe hyperglycaemia. Additionally, individuals with renal impairment indicated with an eGFR of less than 45 ml/min per 1.73 m^2^ were excluded, as SGLT2i were not licensed for use below this threshold for the majority of the study period. Further exclusion criteria are summarised in Fig. 1. Our cohort was split into a population aged less than 70 years at treatment initiation and an older population (≥70 years).

Approval for the study was granted by the CPRD Independent Scientific Advisory Committee (ISCA 22_002000).

### Outcomes

AEs included in the analysis were genital infections, micturition control (urge incontinence, urgency, stress incontinence or nocturnal enuresis), volume depletion and dehydration, urinary frequency, falls, lower limb amputation and DKA. The occurrence of each AE was measured up to 3 years after treatment initiation and censoring of the follow-up time was implemented in case of a discontinuation of the study treatment or start of the comparison study treatment. Individuals were therefore followed up until the earliest of: date of the outcome of interest, discontinuation of the study treatment, start of comparison study treatment, date of practice deregistration/death, end of study period, or 3 years. Occurrences of AEs were identified using diagnosis code lists published at: https://github.com/Exeter-Diabetes/CPRD-Codelists. Genital infections were identified with either a diagnosis code for a specific genital infection (e.g. candida vaginitis or vulvo-vaginitis in women, balanitis, balanoposthitis in men), a prescription for antifungal therapy used specifically to treat genital infections (e.g. an antifungal vaginal pessary), or a non-specific diagnosis of ‘thrush’ with a topical antifungal prescribed on the same day [24]. The diagnosis codes to identify amputation AEs were taken from Pearson-Stuttard et al [25]. DKA was identified using HES hospitalisation data. Treatment outcomes to assess relative effectiveness of SGLT2i included achieved HbA_1c_ (in mmol/mol and %) and weight (kg) on unchanged therapy. These outcome measurements were taken as the closest recorded value to 12 months post-treatment initiation, within a window of 3 to 15 months.

### Covariates

Measured covariates for all outcome models were extracted following our previous protocol [23] together with general information about individuals, including sociodemographic features (age, sex, ethnicity and deprivation) and treatment history, important biomarkers as well as history of relevant comorbidities. Biomarker baseline values are defined nearest to treatment initiation up to 2 years before and 7 days after initiation. Initiation of relevant additional treatments, such as diuretics, have been observed up to 3 months before treatment initiation and comorbidities have been characterised to be within 1 year, 1–5 years or >5 years to treatment initiation. A summary of all covariates is given in Table 1; a cohort description and a comprehensive overview of the biomarker and comorbidity definitions are given here: https://github.com/Exeter-Diabetes/CPRD-Cohort-scripts.

**Table 1:** Baseline characteristics of the study cohort

Variable	SGLT2i <70 years (*n*=66,810)	SGLT2i ≥70 years (*n*=10,419)	DPP4i <70 years (*n*=76,172)	DPP4i ≥70 years (*n*=33,434)
Age, years	55.8 (8.83)	74.5 (3.81)	56.7 (8.98)	77.3 (5.37)
Sex (%)				
Male	40,863 (61.2)	6344 (60.9)	47,185 (61.9)	18,449 (55.2)
Female	25,947 (38.8)	4075 (39.1)	28,987 (38.1)	14,985 (44.8)
HbA_1c_, mmol/mol	77.6 (15.0)	74.8 (13.8)	74.1 (14.5)	71.0 (12.9)
HbA_1c_, %	9.3 (1.37)	9.0 (1.26)	8.9 (1.33)	8.6 (1.18)
eGFR, ml/min per 1.73 m^2^	97.1 (14.3)	80.4 (12.5)	94.1 (16.4)	73.1 (15.4)
eGFR, ml/min per 1.73 m^2^ (%)				
45–59	548 (0.8)	590 (5.7)	2825 (3.7)	7668 (22.9)
60–89	16,886 (25.3)	6335 (60.8)	21,402 (28.1)	18,687 (55.9)
90+	49,054 (73.4)	3469 (33.3)	51,390 (67.5)	6909 (20.7)
ALT, U/l	35.6 (20.5)	27.6 (15.2)	34.8 (20.5)	24.9 (14.6)
BMI, kg/m^2^	34.2 (6.9)	31.6 (5.8)	32.7 (6.8)	30.0 (5.6)
Weight, kg	98.9 (22.1)	89.2 (18.3)	94.1 (21.4)	83.3 (17.5)
Insulin ever taken (%)	9326 (14.0)	1365 (13.1)	3300 (4.3)	2011 (6.0)
T2D duration, years	9.33 (6.07)	13.2 (6.99)	7.77 (5.7)	11.8 (7.4)
DPP4I type (%)				
Alogliptin			15,088 (19.8)	6901 (20.6)
Linagliptin			14,657 (19.2)	10,820 (32.3)
Saxagliptin			4507 (5.9)	1725 (5.2)
Sitagliptin			41,281 (54.2)	13,717 (41.0)
Vildagliptin			639 (0.8)	271 (0.8)
SGLT2Ii type (%)				
Canagliflozin	11,307 (16.9)	2177 (20.9)		
Dapagliflozin	30,253 (45.3)	3701 (35.5)		
Empagliflozin	25,181 (37.7)	4524 (43.4)		
Ertugliflozin	69 (0.1)	17 (0.2)		
Number of concurrent T2D treatments (%)				
1	3554 (5.3)	739 (7.1)	5877 (7.7)	5375 (16.1)
2	29,891 (44.7)	3892 (37.4)	45,043 (59.1)	18,475 (55.3)
3+	33,365 (49.9)	5788 (55.6)	25,252 (33.2)	9584 (28.7)
Number of T2D treatments ever (%)				
1	523 (0.8)	48 (0.5)	1404 (1.8)	1057 (3.2)
2	13,346 (20.0)	1282 (12.3)	32,001 (42.0)	11,886 (35.6)
3	18,475 (27.7)	2566 (24.6)	30,650 (40.2)	13,847 (41.4)
4+	34,466 (51.6)	6523 (62.6)	12,117 (15.9)	6644 (19.9)
Year of treatment initiation (%)				
2013	1127 (1.7)	127 (1.2)	9305 (12.2)	3345 (10.0)
2014	4971 (7.4)	566 (5.4)	9499 (12.5)	3539 (10.6)
2015	8910 (13.3)	1245 (11.9)	10,542 (13.8)	4290 (12.8)
2016	9805 (14.7)	1316 (12.6)	11,745 (15.4)	4959 (14.8)
2017	10,904 (16.3)	1494 (14.3)	11,659 (15.3)	5300 (15.9)
2018	12,271 (18.4)	2054 (19.7)	11,016 (14.5)	5310 (15.9)
2019	13,320 (19.9)	2542 (24.4)	9059 (11.9)	4910 (14.7)
2020	5502 (8.2)	1075 (10.3)	3347 (4.4)	1781 (5.3)
Ethnicity (%)				
White	50,321 (75.3)	9072 (87.1)	55,279 (72.6)	28,787 (86.1)
South Asian	10,172 (15.2)	791 (7.6)	12,576 (16.5)	2450 (7.3)
Black	3086 (4.6)	266 (2.6)	4580 (6.0)	1342 (4.0)
Other	1041 (1.6)	107 (1)	1348 (1.8)	299 (0.9)
Mixed	722 (1.1)	53 (0.5)	863 (1.1)	200 (0.6)
Deprivation index (%)				
1–2	10,603 (15.9)	2338 (22.5)	10,772 (14.1)	7118 (21.3)
3–4	11,380 (17.0)	2274 (21.8)	12,622 (16.6)	7001 (20.9)
5–6	12,780 (19.1)	2033 (19.5)	14,197 (18.6)	6809 (20.4)
7–8	15,272 (22.9)	2003 (19.2)	17,958 (23.6)	6622 (19.8)
9–10	16,736 (25.1)	1765 (16.9)	20,583 (27.0)	5861 (17.5)
Smoking status				
Active smoker	11,793 (17.7)	951 (9.1)	14,803 (19.4)	2968 (8.9)
Ex-smoker	35,054 (52.5)	6806 (65.3)	37,892 (49.7)	20,718 (62)
Non-smoker	17,275 (25.9)	2176 (20.9)	19,927 (26.2)	7930 (23.7)
Medication use (%)				
Loop diuretics	2428 (3.6)	997 (9.6)	3288 (4.3)	4836 (14.5)
Potassium-sparing diuretics	1185 (1.8)	314 (3.0)	1507 (2.0)	1298 (3.9)
Thiazide diuretics	7730 (11.6)	1772 (17)	9312 (12.2)	5916 (17.7)
Immunosuppressants	625 (0.9)	144 (1.4)	838 (1.1)	428 (1.3)
Oestrogens	853 (1.3)	69 (0.7)	950 (1.2)	314 (0.9)
Oral steroids	1579 (2.4)	454 (4.4)	2274 (3.0)	1993 (6.0)
Statins	48,595 (72.7)	8132 (78.0)	54,851 (72)	25,313 (75.7)
ACE inhibitors	28,655 (42.9)	4714 (45.2)	31,242 (41)	14,529 (43.5)
Comorbidities (%)				
Genital infection	34,577 (51.8)	5277 (50.6)	36,903 (48.4)	16,432 (49.1)
Urinary frequency	6530 (9.8)	1638 (15.7)	7499 (9.8)	5365 (16.0)
Micturition control	6002 (9.0)	1247 (12.0)	6866 (9.0)	5059 (15.1)
Volume depletion	5630 (8.4)	1147 (11)	6369 (8.4)	4548 (13.6)
Benign prostatic hyperplasia	2200 (3.3)	1448 (13.9)	2963 (3.9)	5057 (15.1)
Lower limb fractures	4650 (7.0)	851 (8.2)	4948 (6.5)	3061 (9.2)
Falls	7907 (11.8)	2376 (22.8)	8921 (11.7)	9300 (27.8)
Amputation	333 (0.5)	51 (0.5)	415 (0.5)	282 (0.8)
DKA	431 (0.6)	31 (0.3)	367 (0.5)	166 (0.5)
Dementia	153 (0.2)	189 (1.8)	274 (0.4)	1674 (5.0)
Cancer	3833 (5.7)	1653 (15.9)	5160 (6.8)	6415 (19.2)
Asthma	13,678 (20.5)	1962 (18.8)	14,372 (18.9)	6247 (18.7)
COPD	3684 (5.5)	1223 (11.7)	4692 (6.2)	4411 (13.2)
Heart failure	2437 (3.6)	907 (8.7)	3169 (4.2)	4141 (12.4)
CVD^a^	13,131 (19.7)	3841 (36.9)	15,349 (20.2)	14,067 (42.1)
Myocardial infarction	4290 (6.4)	1267 (12.2)	4998 (6.6)	4430 (13.3)
Stroke	2334 (3.5)	816 (7.8)	3145 (4.1)	3806 (11.4)
Revascularisation	4298 (6.4)	1271 (12.2)	4929 (6.5)	4135 (12.4)
Ischaemic heart disease	8320 (12.5)	2605 (25.0)	9787 (12.8)	9382 (28.1)
Angina	6141 (9.2)	1984 (19.0)	7181 (9.4)	6880 (20.6)
Peripheral arterial disease	3040 (4.6)	872 (8.4)	3548 (4.7)	3692 (11.0)
Transient ischaemic attack	1346 (2.0)	578 (5.5)	1668 (2.2)	2514 (7.5)
Chronic liver disease	8366 (12.5)	959 (9.2)	8093 (10.6)	2243 (6.7)
Osteoporosis	666 (1.0)	384 (3.7)	924 (1.2)	1920 (5.7)

Values for continuous variables are given as mean ± SD and binary and categorical variables as *n* (%)

^a^CVD: composite of myocardial infarction, stroke, revascularisation, ischaemic heart disease, angina, peripheral arterial disease, transient ischaemic attack

ALT, alanine aminotransferase; COPD, chronic obstructive pulmonary disease; T2D, type 2 diabetes

### Statistical methods

#### Causal analysis

When analysing treatment effects from observational data, bias due to confounding by indication is a major challenge. The confounding pre-treatment variables affect the outcome and the treatment decision simultaneously. As a result, it is possible that they differ in distribution between individuals who received the study treatment and comparator treatment [26]. Traditional methods such as propensity score matching can mitigate the risk of bias by adjusting for measured confounders, but they cannot control for variables that are not recorded in the data, which can lead to unmeasured confounding [26]. With the instrumental variable approach and given a suitable instrument, treatment effects can be estimated in the presence of residual or unmeasured confounding without bias [27]. The basic idea of the instrumental variable approach is that a suitable instrumental variable is used to extract variation of the treatment that is free of unmeasured confounding. This variation is then used to estimate the treatment effect [26]. We employ the advanced instrumental variable approach proposed by Ertefaie et al [28] which makes use of observed treatment behaviour and covariates to construct a proxy for prescription preference. Importantly, the method is capable of estimating the treatment effect without bias even in the presence of non-ignorable missingness in covariates. Our analysis did therefore not rely on a possibly selective complete case dataset. A more detailed explanation of this approach and a description of the assumed data structure for this study can be found in electronic supplementary material (ESM) Methods and ESM Fig. 1.

All binary AE outcomes were modelled using generalised Poisson regression with follow-up time (in days) as offset. For the estimation of the treatment effect of SGLT2i on achieved HbA_1c_ and weight, a linear outcome model was used. Models used in the instrumental variable estimation and for all outcomes of interest were adjusted using different sets of relevant covariates specific to each outcome. A summary of all models is provided in ESM Table 1.

#### Sensitivity analysis

We performed the following sensitivity analyses to assess the robustness of our findings: (1) To increase power due to the low number of events for several outcomes, we defined additional composite outcomes of osmotic symptoms (comprising volume depletion/dehydration, micturition control and urinary frequency) and combined falls and lower limb fracture (as not all falls might be coded in the CPRD data and lower limb fractures are often caused by falls). Our code list for lower limb fractures excludes fractures of the foot but includes hip fractures, of which 98% are caused by a fall [29]; (2) We additionally censored individuals who switched or added any other type 2 diabetes treatments, other than the study treatments, over follow-up; (3) We repeated the analysis using 1 year maximum follow-up time for AE outcomes to assess short term risks; and (4) We excluded the second drug exposure period for individuals who initiated both treatments over the study period.

## Results

The study cohort included 186,835 episodes of participants commencing treatment with SGLT2i or DPP4i from 161,825 individuals (25,010 initiated both treatments) (Fig. 1). There were 142,982 episodes included in the analysis for adults under 70 (<70) (*n*=66,810 SGLT2i, *n*=76,172 DPP4i) and 43,853 episodes for adults 70 and older (≥70) (*n*=10,419 SGLT2i, *n*=33,434 DPP4i). Table 1 shows the baseline characteristics of the study population by treatment arm and age group. In the supplementary material a more detailed summary of comorbidity history is provided (ESM Table 2) as well as a summary of the amount of missing data for each clinical characteristic (ESM Table 3).

**Fig. 2:**
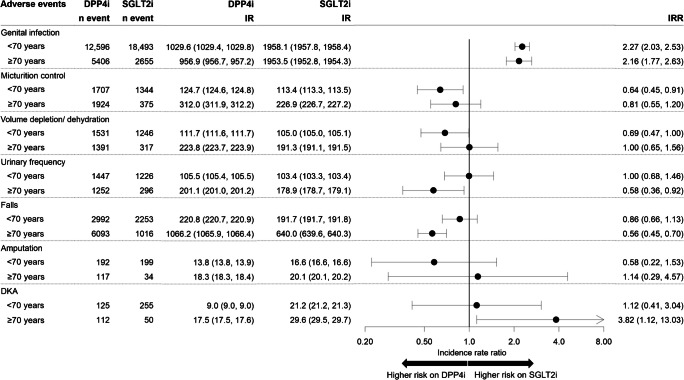
Causal effect estimation results of the IRR for AEs estimated for *n*=142,982 participants <70 (*n*=66,810 SGLT2i, *n*=76,172 DPP4i) and *n*=43,853 participants ≥70 (*n*=10,419 SGLT2i, *n*=33,434 DPP4i). Additionally, the figure shows number (*n*) of events recorded and IRs per 10,000 person-years. Values in brackets represent 95% CIs

Incidence rate ratio (IRR) estimates for each AE of interest are reported in Fig. 2, and person-years and mean follow-up time for all AEs are reported in ESM Table 4.

### Risk of genital infection for people with type 2 diabetes initiating SGLT2i is similarly increased in adults under and over 70

Genital infections were the most commonly recorded AE (Fig. 2), with the highest incidence in adults ≥70 commencing treatment with SGLT2i (SGLT2i incidence rate [IR] 1953.5 [95% CI 1952.8, 1954.3] per 10,000 person-years; DPP4i 956.9 [956.7, 957.2]). Causal treatment estimates suggested SGLT2i were associated with a 2.16 (1.77, 2.63) IRR of genital infection compared with DPP4i in adults ≥70, with a similar IRR in adults under 70 (2.27 [2.03, 2.53].

### DKA is a rare AE and the risk increase with SGLT2i may be restricted to adults over 70

DKA was a rare event, and the highest IR was recorded for individuals ≥70 on SGTL2i (SGLT2i IR 29.6 [95% CI 29.5, 29.7] per 10,000 person-years; DPP4i IR 17.5 [17.5, 17.6]). Causal estimates suggested IRR for DKA with SGLT2i (compared with DPP4i) was increased for those ≥70 (IRR 3.82 [1.12, 13.03]), but not those under 70 (IRR 1.12 [0.41, 3.04]) (Fig. 2).

### Risk of osmotic AE is not increased with SGLT2i in adults under and over 70

IRs for the AE micturition control for those ≥70 taking SGLT2i was 226.6 [95% CI 226.7, 227.2] per 10,000 person-years and the causal estimates did not show an increased risk in this patient group compared with people taking DPP4i (IRR 0.81 [0.55, 1.20]). For the AE volume depletion (including dehydration), IRs in those ≥70 taking SGLT2i were 191.3 [191.1, 191.5] per 10,000 person-years. Causal estimates of risk are not increased for this group (IRR 1.00 [0.65, 1.56]). Additionally, the IR of the AE urinary frequency was 178.9 [178.7, 179.1] per 10,000 person-years and no increased risk was found for those ≥70 taking SGLT2i compared with those taking DPP4i (IRR 0.58 [0.36, 0.92]) from the causal analysis.

### Risk of falls and amputations is not increased with SGLT2i in adults under and over 70

The highest IR for falls was recorded for those ≥70 (SGLT2i IR 640.0 [95% CI 639.6, 640.3] per 10,000 person-years; DPP4i 1066.2 [1065.9, 1066.4]). Results of the causal analysis did not show evidence of an increased IRR of falls for SGLT2i in comparison with DPP4i treatment (IRR 0.86 [0.66, 1.13] for those <70 and 0.56 [0.45, 0.70] for those ≥70) (Fig. 2).

Lower limb amputation was rare and a higher IR was recorded for those ≥70 (SGLT2i incident rate 20.1 [95% CI 20.1, 20.2] per 10,000 person-years; DPP4i 18.3 [18.3, 18.4]). In causal analysis, there was no evidence of an increased risk of lower limb amputation (IRR 0.58 [0.22, 1.53] for those <70; 1.14 [0.29, 4.57] for those ≥70) (Fig. 2).

### Glucose-lowering efficacy of SGLT2i is similar to DPP4i in older people, but in younger adults SGLT2i are more effective

**Fig. 3:**
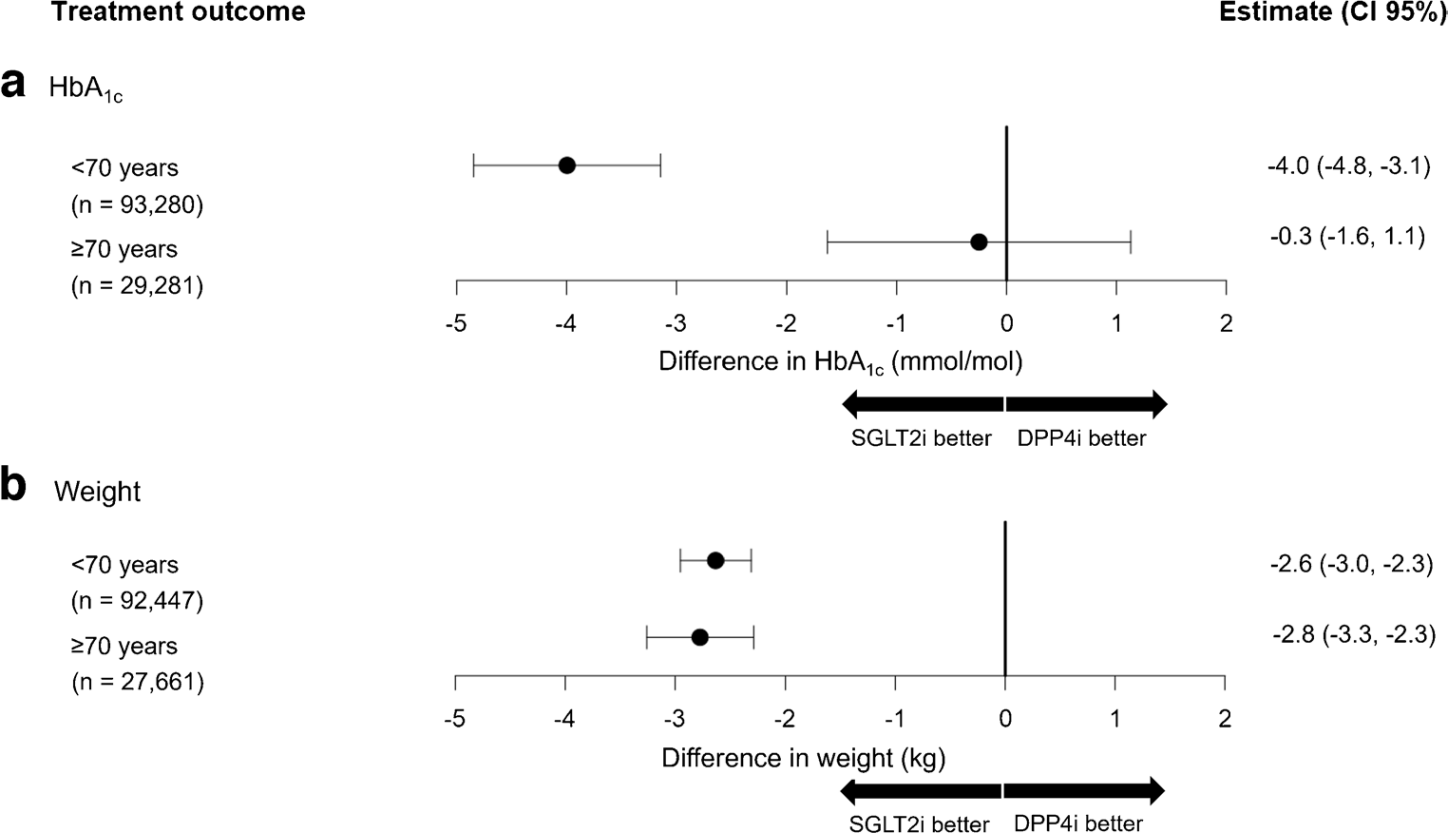
Causal effect estimation results for change in HbA_1c_ (mmol/mol) (**a**) and weight (kg) (**b**). Point estimates represent the difference in outcome with SGLT2i compared to DPP4i, with negative values representing a greater HbA_1c_/weight reduction with SGLT2i over DPP4i. *n* values represent the cases with valid outcome value for which the complete case analysis is applied

Unadjusted mean HbA_1c_ response for those <70 was −12.3 mmol/mol [95% CI −12.4, −12.1] (−1.1% [−1.1, −1.1]) on SGLT2i and −7.7 mmol/mol [−7.8, −7.5] (−0.7% [−0.7, −0.7]) in those taking DPP4i. For those ≥70, unadjusted HbA_1c_ response was −9.9 mmol/mol [−10.2, −9.5] (−0.9% [−0.9, −0.9]) on SGLT2i and −8.5 mmol/mol [−8.7, −8.4] (−0.8% [−0.8, −0.8]) on DPP4i. Causal estimates for differences in HbA_1c_ response and weight change between therapies are shown in Fig. 3. For those <70, there was a greater reduction in HbA_1c_ with SGLT2i compared with DPP4i of −4 mmol/mol [−4.8, −3.1] (−0.4% [−0.4, −0.3]). For those ≥70, HbA_1c_ response on both drug classes was similar (HbA_1c_ differences between therapies −0.25 mmol/mol [−1.63, 1.13], −0.02% [−0.1, 0.1], favouring SGLT2i). In contrast, the causal analysis results show a greater reduction in weight with SGLT2i compared with DPP4i in both age groups, with an SGLT2i benefit of −2.6 kg [−3.0, −2.3] for those <70 and −2.8 kg [−3.3, −2.3] for those ≥70. Unadjusted mean weight response was higher for participants initiating SGLT2i, with −3.9 kg [−4.0, −3.8] for those <70 and initiating SGLT2i and −1.1 kg [−1.1, −1.1] on DPP4i, respectively. For those ≥70, unadjusted weight response was −4.1 kg [−4.2, −4.0] on SGLT2i and −1.3 kg [−1.3, −1.2] on DPP4i.

### Results of the sensitivity analysis are consistent with the main causal analysis results

Results of all sensitivity analyses are given in ESM Table 5. Results were similar to the primary analysis when: (1) using composite outcomes for osmotic symptoms and falls/lower limb fractures; (2) censoring follow-up time at any change in treatment regimen; (3) restricting maximum follow-up time post-drug initiation to one year (except that DKA risk in those ≥70 was no longer significantly increased); and (4) excluding individuals initiating both treatments over the study period.

## Discussion

Our large-scale causal analysis provides important real-world evidence supporting careful use of SGLT2i in older adults. Importantly, we found no increased risk of falls, osmotic symptoms or amputations in those over 70. AEs of potential concern were genital infections and, rarely, DKA. We also demonstrate that SGLT2i are effective in reducing HbA_1c_ in this age group, although the substantially greater glucose-lowering effect than DPP4i in younger adults with this agent is absent in the elderly, where both agents had similar efficacy.

Risk of genital infections was increased in individuals taking SGLT2i to a similar degree in both those under and over 70. This finding complements similar findings in previous meta-analysis [30] and observational data [24], which did not specifically evaluate risk in older adults. Although we found DKA risk with SGLT2i was elevated in those over 70, incidence was very low. This finding supports the warnings of the FDA [15] and the EMA [16] and stresses the need to take DKA risk factors into account when prescribing SGLT2i to older people [11, 17].

A greater mean glycaemic efficacy with SGLT2i compared with DPP4i has been consistently shown in previous RCTs [31, 32], meta-analyses [33] and observational data [34] which did not specifically evaluate older adults. We identify heterogeneity in relative glycaemic efficacy, with greater efficacy in those <70 but not in those ≥70. This lack of glycaemic benefit with SGLT2i in older adults may relate to the association between increasing age and lower eGFR, a known predictor of attenuated glycaemic response with SGLT2i [35]. Weight reduction after SGLT2i initiation is confirmed from our results for both age-stratified populations. Previous RCT meta-analysis results comparing SGLT2i and DPP4i showed a greater weight reduction with SGLT2i of −2.45 kg [95% CI −2.71, −2.19] [5]. The extent of weight reduction in our study is similar to these results.

A major strength of our causal analysis lies in the application of the advanced instrumental variable method by Ertefaie et al [28], which addresses possible unmeasured confounding and does not rely on complete case analysis due to missingness in measured baseline characteristics. The analysis was conducted with a large real-world primary care dataset linked to hospitalisation data, capturing a broad range of AEs for SGLT2i with comprehensive primary and secondary care data.

Limitations of this study are that the analysis relies on correct clinical coding of the AEs, which can be subject to inaccuracies due to miscoding or non-coding. For example, some under-representation of genital infections might be possible as antifungal medication is available as an over-the-counter medication and can be treated without having presented to primary care. Additionally, information about the severity of the AEs was not available [24]. A limitation of the instrumental variable method is that some of the data structure assumptions made are not testable with the data. Additionally, as prescription preference was not measured in the data, our analysis relies on a proxy measurement, which might be subject to measurement errors. Previous similar instrumental variable analyses assessing relative effectiveness and risk of type 2 diabetes treatments in the CPRD data have found that the instrumental variable assumptions are reasonable in this setting [36, 37].

## Conclusion

SGLT2i in older adults are effective and do not increase risk of dehydration, falls or urinary problems in older adults with type 2 diabetes. However, risk of genital infections is increased, and DKA is a rare but severe AE of concern, meaning baseline DKA risk should be carefully assessed before initiation of SGLT2i. This study provides a valuable causal analysis framework for the study of older adults who are generally not included in randomised controlled trials.

## Supplementary Information

Below is the link to the electronic supplementary material.

ESM (PDF 401 KB)

## Data Availability

CPRD data are available by application to the CPRD Independent Scientific Advisory Committee. R code to preproduce the analysis in this paper is available at https://github.com/Exeter-Diabetes/CPRD-Laura-SGLT2i-in-older-adults.

## Abbreviations

AE: Adverse event
CPRD: Clinical Practice Research Datalink
DKA: Diabetic ketoacidosis
DPP4i: Dipeptidyl peptidase-4 inhibitors
EMA: European Medicines Agency
FDA: U.S. Food and Drug Administration
HES: Hospital Episode Statistics
IR: Incidence rate
IRR: Incidence rate ratio
SGLT2i: Sodium-glucose cotransporters 2 inhibitors
