# Supplementary material for: Safety and effectiveness of SGLT2 inhibitors in a UK population with type 2 diabetes and aged over 70 years: an instrumental variable approach
Source: Diabetologia. 2024 Jun 5;67(9):1817–27. doi: 10.1007/s00125-024-06190-9 (PMC11410842; doi:10.1007/s00125-024-06190-9)
Supplement: Supplementary file 1 — ESM (PDF 401 KB) [file 125_2024_6190_MOESM1_ESM.pdf]

Electronic supplementary material

ESM Methods

ESM Figure 1 represents the assumed data structure of this observational study that are pertinent to the instrumental variable (IV) analysis. Arrows in the graph represent assumed causal relationships between the variables. The aim of the study is to estimate the causal effect of receiving SGLT2 inhibitors versus DPP4 inhibitors on the outcome(s) of interest. In particular, we assume that provider prescription preference is a suitable IV and fulfils the IV assumptions, conditional on a set of measured confounders, W. The IV assumptions are: (1) The IV must be strongly associated with the exposure given W, (2) be independent of unmeasured confounders given W and (3) not have a direct effect on the outcome of interest given W [1]. Necessary conditions for the IV assumptions to hold are that (1) between-provider variation in the use of study treatment exists, (2) individuals selection/assignment to a provider is unrelated to providers' preference of the study treatment, (3) a providers' use of treatment is independent of the use of alternative treatments that affect the outcome of interest. [2, 3].

As provider prescription preference is not directly measured in CPRD, and the set of measured confounders contains missing data, we use a proxy variable for it following the approach of Ertefaie et al. [4].

The following non-technical description aims to give interested readers a better understanding of the steps necessary for the construction of a proxy variable for provider prescription preference and the estimation of the causal treatment effects.

For a more in depth and mathematical description of the methods, we refer to the original paper by Ertefaie et al. [4].

The Ertefaie IV approach is conducted in two steps. To apply this approach the measured confounders/ covariates are grouped into:

- W_{obs}: all measured confounders that are observed for all individuals in the data and
- W_{miss}: all measured confounders with at least one missing data point.

Step 1 of the method aims to construct a binary proxy IV for provider prescription preference which will be used as instrument, taking the value 1 when a provider has a preference for SGLT2 inhibitors over DPP4 inhibitors, and 0 otherwise. It is constructed using a generalized mixed effect model for the treatment decision adjusted for all measured confounders (W_{obs} and W_{miss}). The model is estimated using a complete case dataset (i.e. for individuals with complete information on W_{obs} and W_{miss}) and with a random intercept for provider. From this model the fitted values of the random intercept and its empirical distribution is used for the construction of the instrument. The instrument will take on the value 1 for each provider with an estimated random intercept larger than the median of all estimated random intercepts, and 0 otherwise. Please note that the instrument can only be calculated for provider with at least one measured covariate completely measured (W_{obs}). If this is not the case, the provider will needs to be excluded from the IV analysis.

The second step of the Ertefaie method includes the calculation of the causal treatment effect with the Two-Stage Least Squares approach for continuous outcomes and the Two Stage Predictor Substitution method otherwise. [1] This estimation step is applied to all individuals in the dataset, but with adjustment for W_{obs} only.

Specifically, in stage 1, a logit model for the observed treatment decision is fitted which adjusts for W_{obs} and the instrument. Thereafter, in stage 2, the outcome is regressed on the predicted treatment decision and W_{obs} to estimate the causal treatment effect. For the continuous treatment outcomes, achieved HbA_{1c} and weight, a linear outcome model is estimated. In case of binary adverse event outcomes, we used a Poisson model with follow-up time (in days) as offset.

ESM Tables

ESM Table 1

	**Achieved HbA_{1c}**	**Achieved weight**	**Genital infections**	**Osmotic symptoms**	**Falls**	**Lower limb amputations**	**Amputations**	**Diabetic ketoacidosis**
**General characteristics and treatment regime information**	Age, sex, ethnicity, deprivation index, smoking status, diabetes duration, year of study treatment initiation, line of therapy, number of concurrent treatments, insulin taken							
**Biomarkers**	HbA_{1c}, eGFR, BMI/ weight, ALT							
**History of comorbidities**			Genital infections	Osmotic symptom, benign prostate hyperplasia	Oestrogens, oral steroids, statins, Ksparing, loops, thiazide diuretics, ACE inhibitors			
**Additional treatments**			Immuno-suppressants, oestrogens, oral steroids	Ksparing, Loops, thiazide diuretics	lower limb fracture, falls, amputation, Diabetic ketoacidosis, Dementia, Cancer, Asthma, COPD*, heart failure, CVD †, CLD ‡, osteoporosis			

ESM Table 1: Summary of the relevant covariates for each outcome of interest used in the IV estimation. For the analysis of achieved weight, baseline weight instead of baseline BMI was included. Models of the first step of IV method by Ertefaie et al. 2017 and the first stage regression model of the IV estimation adjusted for all relevant covariates, whereas the outcome models only included completely recorded covariates. *COPD: chronic obstructive pulmonary disease, †CVD: composite of myocardial infarction, stoke, revascularisation, ischemic heart disease, angina, peripheral arterial disease, transient ischemic attack, ‡CLD: chronic liver disease.

ESM Table 2

	SGLT2i <70 years (n = 66810)	SGLT2i ≥70 years (n = 10419)	DPP4i <70 years (n = 76172)	DPP4i ≥70 years (n = 33434)
**Genital infection**				
Never	32,233 (48.2)	5142 (49.4)	39,269 (51.6)	17,002 (50.9)
<1 year	8950 (13.4)	1197 (11.5)	10,566 (13.9)	3920 (11.7)
1-5 years	13,114 (19.6)	1890 (18.1)	13,717 (18)	5610 (16.8)
>5 years	12,513 (18.7)	2190 (21)	12,620 (16.6)	6902 (20.6)
**Urinary frequency**				
Never	60,280 (90.2)	8781 (84.3)	68,673 (90.2)	28,069 (84)
<1 year	819 (1.2)	178 (1.7)	1170 (1.5)	832 (2.5)
1-5 years	2309 (3.5)	598 (5.7)	2802 (3.7)	1969 (5.9)
>5 years	3402 (5.1)	862 (8.3)	3527 (4.6)	2564 (7.7)
**Micturition control**				
Never	60,808 (91)	9172 (88)	69,306 (91)	28,375 (84.9)
<1 year	786 (1.2)	180 (1.7)	1087 (1.4)	1024 (3.1)
1-5 years	2024 (3)	425 (4.1)	2340 (3.1)	1732 (5.2)
>5 years	3192 (4.8)	642 (6.2)	3439 (4.5)	2303 (6.9)
**Volume depletion**				
Never	61,180 (91.6)	9272 (89)	69,803 (91.6)	28,886 (86.4)
<1 year	730 (1.1)	164 (1.6)	960 (1.3)	815 (2.4)
1-5 years	1881 (2.8)	398 (3.8)	2173 (2.9)	1551 (4.6)
>5 years	3019 (4.5)	585 (5.6)	3236 (4.2)	2182 (6.5)
**Falls**				
Never	58,903 (88.2)	8043 (77.2)	67,251 (88.3)	24,134 (72.2)
<1 year	1128 (1.7)	603 (5.8)	1473 (1.9)	2989 (8.9)
1-5 years	2738 (4.1)	902 (8.7)	3224 (4.2)	3634 (10.9)
>5 years	4041 (6)	871 (8.4)	4224 (5.5)	2677 (8.0)
**Amputation**				
Never	66,477 (99.5)	10,368 (99.5)	75,757 (99.5)	33,152 (99.2)
<1 year	75 (0.1)	6 (0.1)	111 (0.1)	62 (0.2)
1-5 years	147 (0.2)	20 (0.2)	182 (0.2)	115 (0.3)
>5 years	111 (0.2)	25 (0.2)	122 (0.2)	105 (0.3)
**Diabetic ketoacidosis**				
Never	66,379 (99.4)	10,388 (99.7)	75,805 (99.5)	33,268 (99.5)
<1 year	61 (0.1)	6 (0.1)	95 (0.1)	66 (0.2)
1-5 years	173 (0.3)	15 (0.1)	129 (0.2)	48 (0.1%)
>5 years	197 (0.3)	10 (0.1)	143 (0.2)	52 (0.2)

ESM Table 2: Detailed description of potential recurrent comorbidities in the study population recorded prior to study treatment initiation.

ESM Table 3

	SGLT2i <70 years (n = 66,810)	SGLT2i ≥70 years (n = 10,419)	DPP4i <70 years (n = 76,172)	DPP4i ≥70 years (n = 33,434)
**HbA_{1c} (mmol/mol and %)**	8973 (13.4)	1406 (13.5)	7559 (9.9)	3999 (12)
**eGFR (ml/min/1.73m^2)**	322 (0.5)	25 (0.2)	555 (0.7)	170 (0.5)
**ALT (U/L)**	4206 (6.3)	577 (5.5)	5,354 (7.0)	2158 (6.5)
**BMI (kg/m^2)**	2646 (4)	371 (3.6)	3,701 (4.9)	1988 (5.9)
**Weight (kg)**	1496 (2.2)	215 (2.1)	2,370 (3.1)	1440 (4.3)

ESM Table 3: Summary of missing data in baseline characteristics of the study population values are given in absolute frequencies in n (%).

ESM Table 4

	Person-years of follow-up	Mean follow-up time (years)
**Adverse event**		
Genital infections	286,867.4	1.54 (1.03)
Volume depletion	334,448	1.79 (1)
Micturition control	333,607.1	1.79 (1)
Urinary frequency	334,596.2	1.79 (1)
Falls	326,040.4	1.75 (1)
Amputation	339,284.1	1.82 (0.99)
DKA	339,574.1	1.82 (0.99)
**Composite adverse event**		
Osmotic symptoms	329,345.2	1.76 (1)
Falls + lower limb fractures	339,848.6	1.82 (0.99)

ESM Table 4: Person-years of follow-up calculated as the total follow-up time and mean follow-up time (in years) of the adverse events. Values for the mean follow-up time are shown as mean (standard deviation).

ESM Table 5

	Composite outcomes	Censoring scheme	Follow-up time	Second study period
**Treatment outcomes**<br>**Mean relative difference (CI 95%)**				
HbA_{1c} (mmol/mol, %)				
<70 years				-4.3 (-5.1, -3.5), -0.4 (-0.5, -0.3)
≥70 years				-0.3 (-1.8, 1.2), -0.03 (-0.2, 0.1)
Weight (kg)				
<70 years				-2.7 (-2.9, -2.3)
≥70 years				-2.7 (-3.2, -2.2)
**Adverse event outcomes**<br>**IRR (CI 95%)**				
Genital infection				
<70 years		2.42 (2.14, 2.74)	2.55 (2.24, 2.9)	2.11 (1.9, 2.34)
≥70 years		2.16 (1.74, 2.68)	2.0 (1.59, 2.56)	2.04 (1.66, 2.51)
Micturition control				
<70 years		0.69 (0.46, 1.02)	0.72 (0.45, 1.16)	0.73 (0.52, 0.98)
≥70 years		0.91 (0.58, 1.42)	0.91 (0.54, 1.51)	1.03 (0.68, 1.54)
Volume depletion/ dehydration				
<70 years		0.73 (0.48, 1.11)	0.8 (0.49, 1.32)	0.73 (0.52, 1.03)
≥70 years		1.1 (0.67, 1.82)	1.33 (0.74, 2.38)	1.35 (0.85, 2.14)
Urinary Frequency				
<70 years		0.99 (0.64, 1.52)	0.95 (0.57, 1.58)	1.33 (0.94, 1.88)
≥70 years		0.57 (0.33, 0.97)	0.75 (0.4, 1.39)	0.57 (0.34, 0.94)
Osmotic symptoms (composite)				
<70 years	0.81 (0.62, 1.06)			
≥70 years	0.82 (0.6, 1.12)			
Falls				
<70 years		0.83 (0.61, 1.13)	0.89 (0.61, 1.3)	0.95 (0.74, 1.2)
≥70 years		0.62 (0.48, 0.8)	0.46 (0.34, 0.61)	0.62 (0.49, 0.79)
Falls/ lower limb fractions (composite)				
<70 years	0.88 (0.68, 1.13)			
≥70 years	0.59 (0.48, 0.74)			
Amputation				
<70 years		0.91 (0.26, 3.17)	0.22 (0.05, 0.92)	0.84 (0.36, 2.0)
≥70 years		0.72 (0.1, 5.1)	1.4 (0.17, 11.79)	1.08 (0.26, 4.53)

DKA				
<70 years		2.5 (0.37, 17.0)	2.46 (0.54, 11.19)	1.8 (0.72, 4.53)
≥70 years		12.05 (1.6, 91.34)	1.7 (0.3, 9.63)	6.19 (1.78, 21.5)

ESM Table 5: Summary of the sensitivity analysis results. Results for treatment outcomes are given in mean relative difference in outcome measure and AE results as presented as incidence rate ratio. All results are shown with 95% confidence intervals. Composite outcomes: Analysis using a composite for osmotic symptoms as well as falls and lower limb fractures. Censoring scheme: censoring for the AE is done in case of any change of the treatment regime. Follow-up time: Follow-up time of the AEs was maximum 1 year. Second study period: Exclusion of the second study period for individuals who initiated both treatments.

ESM Figures

ESM Figure 1

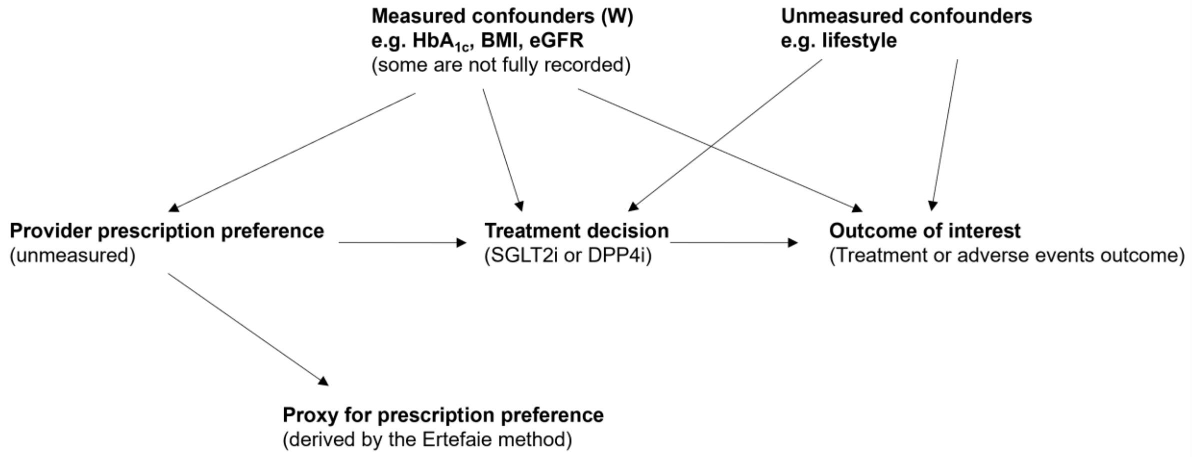

ESM Figure 1: Representation of assumed data structure in this study. Arrows in this plots indicate assumed relationships between variables. The missing of arrows indicates assumed lack of relationship. We assume that the variable provider prescription preference is a valid IV but it is not measured in the data at hand. Therefore, a proxy variable is derived using the IV approach by Ertefaie et al. 2017.

MASTERMIND consortium

Prof Andrew Hattersley[1], Prof Ewan Pearson[2], Dr Angus Jones[1], Dr Beverley Shields[1], Dr John Dennis[1], Dr Lauren Rodgers[1], Prof William Henley[1], Prof Timothy McDonald[1], Prof Michael Weedon[1], Prof Nicky Britten[1], Catherine Angwin[1], Dr Naveed Sattar[3], Dr Robert Lindsay[3], Prof Christopher Jennison[4], Prof Mark Walker[5], Prof Kennedy Cruickshank[6], Dr Salim Janmohamed[7], Prof Christopher Hyde[1], Prof Rury Holman[8], Prof Andrew Farmer[8], Prof Alastair Gray[8], Prof Stephen Gough[8], Dr Olorunsola Agbaje[8], Dr Trevelyan McKinley[1], Dr Sebastian Vollmer[9], Dr Bilal Mateen[7], Prof William Hamilton[1], Dr Katie G. Young[1], Dr Pedro Cardoso[1], Dr Laura Güdemann[1]

[1] University of Exeter

[2] University of Dundee

[3] University of Glasgow

[4] University of Bath

[5] University of Newcastle

[6] Kings College London

[7] University College London

[8] University of Oxford

[9] University of Kaiserslautern

STROBE Statement—checklist of items that should be included in reports of observational studies

	Item No	Recommendation	Page number
**Title and abstract**	1	(*a*) Indicate the study's design with a commonly used term in the title or the abstract	**1**
		(*b*) Provide in the abstract an informative and balanced summary of what was done and what was found	**1**
**Introduction**			
Background/rationale	2	Explain the scientific background and rationale for the investigation being reported	**4-5**
Objectives	3	State specific objectives, including any prespecified hypotheses	**5**
**Methods**			
Study design	4	Present key elements of study design early in the paper	**5**
Setting	5	Describe the setting, locations, and relevant dates, including periods of recruitment, exposure, follow-up, and data collection	**6-8**
Participants	6	(*a*) *Cohort study*—Give the eligibility criteria, and the sources and methods of selection of participants. Describe methods of follow-up<br>*Case-control study*—Give the eligibility criteria, and the sources and methods of case ascertainment and control selection. Give the rationale for the choice of cases and controls<br>*Cross-sectional study*—Give the eligibility criteria, and the sources and methods of selection of participants	**6-8**
		(*b*) *Cohort study*—For matched studies, give matching criteria and number of exposed and unexposed<br>*Case-control study*—For matched studies, give matching criteria and the number of controls per case	
Variables	7	Clearly define all outcomes, exposures, predictors, potential confounders, and effect modifiers. Give diagnostic criteria, if applicable	**6-8**
Data sources/ measurement	8*	For each variable of interest, give sources of data and details of methods of assessment (measurement). Describe comparability of	**6-7**

		assessment methods if there is more than one group	
Bias	9	Describe any efforts to address potential sources of bias	**8-9**
Study size	10	Explain how the study size was arrived at	**5-6**
Quantitative variables	11	Explain how quantitative variables were handled in the analyses. If applicable, describe which groupings were chosen and why	**7-8**
Statistical methods	12	(*a*) Describe all statistical methods, including those used to control for confounding	**8-9**
		(*b*) Describe any methods used to examine subgroups and interactions	**8-9**
		(*c*) Explain how missing data were addressed	**8-9 + Supp. material**
		(*d*) *Cohort study*—If applicable, explain how loss to follow-up was addressed<br>*Case-control study*—If applicable, explain how matching of cases and controls was addressed<br>*Cross-sectional study*—If applicable, describe analytical methods taking account of sampling strategy	
		(*e*) Describe any sensitivity analyses	**9**

Continued on next page

**Results**			
Participants	13*	(a) Report numbers of individuals at each stage of study—eg numbers potentially eligible, examined for eligibility, confirmed eligible, included in the study, completing follow-up, and analysed	**9,10**
		(b) Give reasons for non-participation at each stage	**9, 10+ Figure 1**
		(c) Consider use of a flow diagram	**10 (Figure 1)**
Descriptive data	14*	(a) Give characteristics of study participants (eg demographic, clinical, social) and information on exposures and potential confounders	**10 (Table 1)**
		(b) Indicate number of participants with missing data for each variable of interest	**10 (Supp. Table 3)**
		(c) *Cohort study*—Summarise follow-up time (eg, average and total amount)	**10 (Supp. Table 4)**
Outcome data	15*	*Cohort study*—Report numbers of outcome events or summary measures over time	**10-13 (Figure 2)**
		Case-control study—Report numbers in each exposure category, or summary measures of exposure	

		Cross-sectional study—Report numbers of outcome events or summary measures	
Main results	16	(*a*) Give unadjusted estimates and, if applicable, confounder-adjusted estimates and their precision (eg, 95% confidence interval). Make clear which confounders were adjusted for and why they were included	**10-13 (Figures 2 and 3**
		(*b*) Report category boundaries when continuous variables were categorized	
		(*c*) If relevant, consider translating estimates of relative risk into absolute risk for a meaningful time period	
Other analyses	17	Report other analyses done—eg analyses of subgroups and interactions, and sensitivity analyses	**13**
**Discussion**			
Key results	18	Summarise key results with reference to study objectives	**13-15**
Limitations	19	Discuss limitations of the study, taking into account sources of potential bias or imprecision. Discuss both direction and magnitude of any potential bias	**13-15**
Interpretation	20	Give a cautious overall interpretation of results considering objectives, limitations, multiplicity of analyses, results from similar studies, and other relevant evidence	**13-15**
Generalisability	21	Discuss the generalisability (external validity) of the study results	**13-15**
**Other information**			
Funding	22	Give the source of funding and the role of the funders for the present study and, if applicable, for the original study on which the present article is based	**16**

*Give information separately for cases and controls in case-control studies and, if applicable, for exposed and unexposed groups in cohort and cross-sectional studies.

Note: An Explanation and Elaboration article discusses each checklist item and gives methodological background and published examples of transparent reporting. The STROBE checklist is best used in conjunction with this article (freely available on the Web sites of PLoS Medicine at http://www.plosmedicine.org/, Annals of Internal Medicine at http://www.annals.org/, and Epidemiology at http://www.epidem.com/). Information on the STROBE Initiative is available at www.strobe-statement.org.

ESM Bibliography

[1] M. L. Lousdal, “An introduction to instrumental variable assumptions, validation and estimation,” *Emerg Themes Epidemiol,* vol. 15, no. 1, pp. 1-7, 1 2018.

[2] M. A. Brookhart and S. Schneeweiss, “Preference-based instrumental variable methods for the estimation of treatment effects: assessing validity and interpreting results,” *Int J Biostat,* vol. 3, no. 1, 2007.

[3] E. L. Korn and S. Baumrind, “Clinician Preferences and the Estimation of Causal Treatment Differences,” *Statistical Science,* vol. 13, no. 3, pp. 209-235, 1998.

[4] A. Ertefaie, J. H. Flory, S. Hennessy and D. S. Small, “Instrumental variable methods for continuous outcomes that accommodate nonignorable missing baseline values,” *Am J Epidemiol,* vol. 185, no. 2, pp. 1233-1239, 2017.
